# Mepolizumab therapy improves the most bothersome symptoms in patients with hypereosinophilic syndrome

**DOI:** 10.3389/fmed.2023.1035250

**Published:** 2023-03-29

**Authors:** Florence Roufosse, Joseph Butterfield, Jonathan Steinfeld, Jane H. Bentley, Robyn von Maltzahn, Namhee Kwon, Linda Nelsen

**Affiliations:** ^1^Department of Internal Medicine, Hôpital Erasme, Université Libre de Bruxelles, Brussels, Belgium; ^2^Division of Allergic Diseases and the Mayo Clinic Program for Mast Cell and Eosinophil Disorders, Mayo Clinic, Rochester, MN, United States; ^3^Respiratory Research & Development, GSK, Collegeville, PA, United States; ^4^Value Evidence and Outcomes, GSK, GSK House, Brentford, Middlesex, United Kingdom; ^5^Respiratory Research & Development, GSK, GSK House, Brentford, Middlesex, United Kingdom; ^6^Value Evidence and Outcomes, GSK, Collegeville, PA, United States

**Keywords:** hypereosinophilic syndrome (HES), daily symptoms, HES-DS, most bothersome, patient-reported, mepolizumab

## Abstract

**Background:**

Hypereosinophilic syndrome (HES) is characterized by persistent elevated blood and/or tissue eosinophil levels and eosinophil-mediated organ damage. Presentation is highly heterogenous; patients may experience symptoms affecting multiple organ systems.

**Objectives:**

To assess the effects of mepolizumab, which targets interleukin-5, on HES-related symptom burden, based on HES daily symptoms (HES-DS) questionnaire data collected during the Phase III (ClinicalTrials.gov ID: NCT02836496) study of mepolizumab in patients with HES.

**Methods:**

Each of the six HES-related symptoms were rated (0–10) daily by patients, recalling worst symptom experience in the prior 24 hours; change from baseline at Week 32 was also calculated for mepolizumab versus placebo.

**Results:**

Mepolizumab versus placebo reduced HES-related symptom burden severity in patients with HES at Week 32. Improvements in the median change from baseline scores were seen across all symptom groups except skin for patients treated with mepolizumab; greatest improvement from baseline was observed for breathing symptoms.

**Conclusion:**

These data highlight the considerable symptom burden associated with HES and further support the clinical benefits of mepolizumab treatment for these patients.

## Introduction

Hypereosinophilic syndrome (HES) is a rare, heterogeneous disorder, characterized by blood hypereosinophilia and increased eosinophil levels or marked deposition of eosinophilic granule proteins in tissue, associated with eosinophil-mediated organ damage (1, 2). Blood hypereosinophilia is defined as persistent blood eosinophil counts >1.5×10^9^/L on at least two occasions (2, 3). While patients typically present with a variety of symptoms, pulmonary, cardiovascular, dermatological, and/or gastrointestinal involvement are frequently reported (1). Treatment options typically include long-term oral corticosteroids (OCS), cytotoxic and/or immunosuppressive agents, which are associated with significant toxicity (4). For patients with myeloproliferative HES (M-HES) who test positive for a Fip1-like 1 (FIP1L1)/platelet-derived growth factor receptor alpha (PDGFRA) rearrangement, the tyrosine kinase inhibitor imatinib should be initiated as first-line therapy (3, 5). Mepolizumab, a humanized monoclonal antibody that targets interleukin-5, has recently been approved for the treatment of patients with HES across multiple regions worldwide (6, 7). A recent Phase III study (GSK ID: 200622; NCT02836496) demonstrated that mepolizumab [300 mg, subcutaneously (SC)] significantly reduced the occurrence of flares in patients with HES compared with placebo (8). The impact of mepolizumab on HES-related symptom severity using the HES daily symptoms (HES-DS) questionnaire was assessed as an exploratory endpoint in this study. Here, we describe analyses of most bothersome symptom (HES-DS score) and HES-DS domain scores.

## Materials and methods

The Phase III study (NCT02836496) has been described elsewhere (8). Briefly, this was a randomized, double-blind, multicenter, placebo-controlled study enrolling patients who were ≥12 years of age with HES ≥6 months, had ≥2 flares in the previous 12 months and a screening blood eosinophil count ≥ 1,000 cells/μL. Patients who tested positive for the FIP1L1-PDGFRA fusion were excluded from the study. Patients had ≥4 weeks stable standard of care HES therapy before randomization (1:1) to mepolizumab (300 mg SC) or placebo administered every 4 weeks, plus existing standard of care HES therapy, for 32 weeks. Any existing standard HES therapy remained the same throughout the 32-week treatment period, unless there was a worsening of symptoms (a flare) that required an increase in therapy. Treatment was returned, where possible, to the original dosing regimen once disease control was regained (8–11).

The HES-DS questionnaire (see Supplementary materials) was developed based on a previous qualitative study assessing the symptoms and the impact of HES on patients’ health-related quality of life, wherein patients described their symptomatic experience according to organ system (12). The HES-DS questionnaire included six domains, each representing a single commonly-reported HES symptom category; abdominal pain/bloating, breathing symptoms (e.g., shortness of breath, wheeze), chills/sweats, muscle/joint pain, nasal/sinus symptoms (e.g., congestion, runny nose), and skin symptoms (e.g., itchiness, rash, hives). At randomization, patients identified up to three domains which they considered most bothersome (Table 1); one patient identified four domains. Subsequently, patients completed ratings for all six domains each evening in an eDiary, recalling the worst symptom experience over the previous 24 hours using an 11-point scale (0 = none, 10 = worst imaginable). For each symptom, baseline and Week 32 change from baseline scores for each patient were calculated using the mean of the daily scores during the previous week. Change from baseline HES-DS score at Week 32 was derived as the mean of change from baseline domain scores at Week 32 for the domains identified by that patient as most bothersome. Patients with a missing value for change from baseline HES-DS score or domain score were included in the non-parametric analysis with the largest (i.e., worst) change from baseline value imputed; distribution of scores were compared between treatment groups using a Wilcoxon rank sum test. Parametric analysis was performed using a repeated measures model, in which missing data were assumed missing at random. Analyses of domain scores in the subgroup of patients reporting the domain as most bothersome at baseline were performed *post hoc*. Further information on statistical methods is provided in the Supplementary materials.

**TABLE 1:** Number and type of most bothersome domains reported at baseline by patients.

	Placebo (*N* = 54)	Mepolizumab 300 mg SC (*N* = 54)	Total (*N* = 108)
**Number of HES-DS domains identified at baseline, *n* (%)**			
1	11 (20)	12 (22)	23 (21)
2	15 (28)	11 (20)	26 (24)
3	27 (50)	31 (57)	58 (54)
4	1 (2)	0 (0)	1 (1)
**Domains reported most bothersome at baseline, *n* (%)**			
Abdominal pain/bloating	24 (44)	16 (30)	40 (37)
Breathing symptoms	30 (56)	30 (56)	60 (56)
Chills/sweats	5 (9)	10 (19)	15 (14)
Muscle/joint pain	20 (37)	24 (44)	44 (41)
Nasal/sinus symptoms	19 (35)	22 (41)	41 (38)
Skin symptoms	28 (52)	25 (46)	53 (49)

Up to three most bothersome domains were identified by patients at randomization (1 patient identified four). Therefore, patient numbers for symptom domains in each treatment arm add up to more than the number of patients enrolled in each treatment arm. Percentage values were rounded to whole numbers.

HES-DS, hypereosinophilic syndrome daily symptoms; SC, subcutaneous.

## Results

**FIGURE 1:**
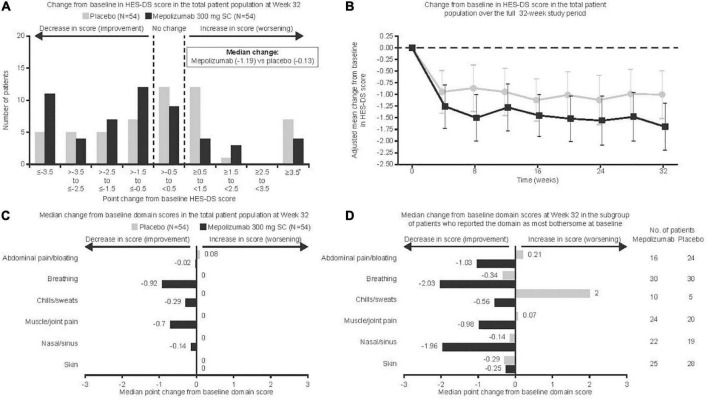
Change from baseline in HES-DS score denotes average change in score for most bothersome domains identified at baseline. Vertical bars **(B)** represent 95% confidence intervals. *Seven placebo and four mepolizumab patients with missing data were included in the worst category (≥3.5) for **(A)**; these data were considered missing at random in **(B)** and were included with the worst change observed for any patient for **(C,D)**. HES-DS, hypereosinophilic syndrome daily symptoms; SC, subcutaneous.

Of the 108 patients enrolled, 54 received mepolizumab and 54 received placebo. Overall, the majority of patients (92%) were receiving HES therapy at baseline, with 72% of patients taking OCS and 21% taking cytotoxic or immunosuppressive therapy; further details have been published previously (8–11). The proportion of patients who experienced disease flares requiring a temporary increase in existing HES therapy has been reported previously (8–11). At baseline, symptom domains were identified as most bothersome by patients with the following frequency; breathing symptoms (56%), skin symptoms (49%), muscle/joint pain (41%), nasal/sinus symptoms (38%), abdominal pain/bloating (37%), and chills/sweats (14%; Table 1). Median (range) baseline HES-DS scores were similar for patients receiving mepolizumab versus placebo [4.18 (0.3, 9.4) vs. 4.37 (0.0, 9.2)]. Mepolizumab was associated with a statistically significant improvement versus placebo in change from baseline HES-DS score at Week 32 (*p* = 0.001, Figure 1A); median change from baseline scores with mepolizumab versus placebo were −1.19 versus −0.13. Seven patients receiving placebo and four receiving mepolizumab had missing data for change from baseline at Week 32 and were included in this non-parametric analysis with the worst change observed for any patient. Parametric analysis demonstrated numerical improvements with mepolizumab versus placebo after the first dose (Week 4; Figure 1B); this improvement was maintained over time. The adjusted mean change from baseline at Week 32 was −0.69 (95% confidence intervals: −1.42, 0.03).

When individual domains were analyzed in the total population, there was an improvement for patients receiving mepolizumab versus placebo in all domains except skin, although the difference for chills/sweats was not significant (*p* = 0.05 for chills/sweats, *p* < 0.05 for all other non-cutaneous symptoms; Figure 1C). No patients in the mepolizumab group experienced a worsening of disease manifestations. Results from the parametric analysis were consistent with the non-parametric analysis, showing a greater reduction in symptom severity score for mepolizumab versus placebo (Table 2). When domain scores were analyzed only in those patients reporting the domain as most bothersome at baseline, there was an improvement for patients receiving mepolizumab versus placebo for breathing symptoms (*p* = 0.01); numerical improvements were observed for all other domains except skin (Figure 1D). Parametric analysis further supported these findings (Table 2).

**TABLE 2:** Difference in mean HES-DS score by domain at Week 32 in the total population and in the subgroup of patients who reported the domain as most bothersome at baseline.

	Number of patients*		Mepolizumab 300 mg SC vs. placebo	*p*-value
	**Mepolizumab 300 mg SC**	**Placebo**		
**HES-DS score, total population, difference in means (95% confidence interval)**				
Abdominal pain/bloating	50	47	−0.70 (−1.39, 0.00)	0.049
Breathing	50	47	−0.91 (−1.68, −0.13)	0.022
Chills/sweats	50	47	−0.78 (−1.47, −0.09)	0.026
Muscle/joint	50	47	−0.76 (−1.52, 0.01)	0.052
Nasal/sinus	50	47	−0.75 (−1.53, 0.03)	0.059
Skin	50	47	−0.25 (−1.04, 0.53)	0.522
**HES-DS score, patients reporting domain as most bothersome, difference in means (95% confidence interval)^†^**				
Abdominal pain/bloating	15	23	−1.18 (−2.43, 0.06)	0.062
Breathing	28	29	−1.44 (−2.56, −0.32)	0.013
Chills/sweats	10	3	−1.75 (−3.74, 0.25)	0.081
Muscle/joint	23	19	−0.91 (−2.37, 0.56)	0.216
Nasal/sinus	21	17	−0.65 (−2.03, 0.73)	0.346
Skin	23	21	0.16 (−1.21, 1.52)	0.815

Parametric analysis was performed using mixed model repeated measures in which missing data was assumed missing at random, with covariates of baseline, baseline OCS dose, region, treatment, and visit, plus interaction terms for visit-by-baseline and visit-by-treatment group.

*Number of patients for which data were analyzed.

^†^Difference in means shown only for patients with available data at Week 32.

HES-DS, hypereosinophilic syndrome daily symptoms; OCS, oral corticosteroids; SC, subcutaneous.

## Discussion

To the best of our knowledge, we are the first to report the daily symptom burden for patients with HES using results from a clinical trial. The patient-centric, individualized nature of the HES-DS tool allowed investigation of the symptoms that mattered most to patients. The most common symptoms at baseline were breathing and skin symptoms, consistent with the most commonly affected organ systems in HES as identified in previous studies (4, 13). The results reported herein suggest that mepolizumab combined with standard of care reduces the severity of HES-related symptoms in patients with HES. Improvements in symptom severity were seen from Week 4 of treatment and when analyzed by individual domains, improvement with mepolizumab versus placebo was seen for all except skin symptoms, although the improvement in symptom severity for chills/sweats was not statistically significant. The greatest improvement from baseline was observed for breathing symptoms, mirroring previous studies of mepolizumab in patients with severe eosinophilic asthma (14).

Although skin was the second most frequently reported domain at baseline, we did not observe significant improvement with mepolizumab using the HES-DS tool. This contrasts with previous studies, potentially reflecting differing delivery/dosing regimens. In a study of intravenous mepolizumab (4-weekly 750 mg), 11/16 patients with skin/subcutaneous manifestations achieved reductions in OCS use (13). In another small study of patients with predominant cutaneous involvement extensive skin lesions regressed following intravenous mepolizumab treatment (4-weekly 750 mg) (15).

While these data further support findings from previous studies indicating the utility of mepolizumab in the treatment of patients with HES, (8, 13, 16) there are several limitations with this study. Firstly, caution is needed when interpreting these results owing to small patient numbers. For example, data reporting chills/sweats as most bothersome only included ten patients receiving mepolizumab and five receiving placebo. Secondly, the global numerical changes do not fully reflect the changes in disease manifestations experienced by patients. Indeed, an improvement in the score relating to a single domain may be clinically meaningful, but its impact may be lost within the total HES-DS score if this is the only disease manifestation that was present at enrollment. Thirdly, most patients were receiving concomitant standard of care therapy during the study that may have impacted symptom severity; however, background treatment was kept stable throughout the course of the study as per protocol therefore minimizing the potential impact. Finally, while derived from qualitative concept elicitation research in patients, (12) the HES-DS questionnaire is not a validated patient-reported outcome tool and does not provide detailed information on various manifestations that may accompany specific organ/tissue involvement. In addition, the minimal clinically important difference in HES-DS score, which reflects an improvement in disease activity, has not been determined. While the development of a clinical score for patients with HES is an unmet need, validation of such tools is difficult due to the intrinsic heterogeneity and rare nature of HES. In conclusion, our results based on patient-reported data support that mepolizumab treatment is of benefit for patients with HES and demonstrates improvement in the majority of symptoms reported most bothersome by patients.

## Data availability statement

The original contributions presented in this study are included in the article/Supplementary material; further enquiries can be submitted via www.clinicalstudydatarequest.com.

## Author contributions

FR, JB, JS, RM, and LN were involved in the conception or design of the work. FR and JHB were involved with the acquisition of data. All authors were involved in drafting the work or revising it critically for important intellectual content (i.e., data analysis and interpretation) and agreed to the submission and to be accountable for all aspects of the work.
