# Supplementary material for: Mepolizumab therapy improves the most bothersome symptoms in patients with hypereosinophilic syndrome
Source: Front Med (Lausanne). 2023 Mar 29;10:1035250. doi: 10.3389/fmed.2023.1035250 (PMC10090544; doi:10.3389/fmed.2023.1035250)
Supplement: Supplementary file 1 [file Data_Sheet_1.docx]

# Supplementary materials

## Mepolizumab therapy improves the most bothersome symptoms in patients with hypereosinophilic syndrome

#### Florence Roufosse, Joseph Butterfield, Jonathan Steinfeld, Jane H. Bentley, Robyn von Maltzahn, Namhee Kwon, Linda Nelsen

## Contents

**Methods**

**HES-DS Questionnaire**

## Methods

The distribution of change from baseline in most bothersome hypereosinophilic syndrome daily symptoms (HES-DS) symptom scores at Week 32 was compared between treatment groups using a Wilcoxon rank sum test stratified by median baseline most bothersome symptom score, baseline oral corticosteroids (OCS) dose (0–≤20 mg/day and >20 mg/day prednisone or equivalent) and region. Parametric analysis was performed using a repeated measures model, in which missing data was assumed missing at random, and was fitted to the HES-DS score at Weeks 4, 8, 12, 16, 20, 24, 28 and 32; model included time point, treatment, and region as fixed categorical effects, baseline HES-DS score and baseline OCS dose as fixed continuous effects and interaction terms for treatment-by-visit and visit-by-baseline score. Similar analyses were conducted for change from baseline domain scores.

## HES-DS Questionnaire

Below are other commonly listed symptoms. Please rate the severity of each symptom today.

1. Please rate your **muscle or joint pain** by marking the one number that best describes your WORST level of muscle pain during the
   past 24 hours.

| **0** | **1** | **2** | **3** | **4** | **5** | **6** | **7** | **8** | **9** | **10** |
| --- | --- | --- | --- | --- | --- | --- | --- | --- | --- | --- |
| **None** |  |  |  |  |  |  |  |  |  | **As bad as you can imagine** |

1. Please rate your **chills or sweats** by marking the one number that best describes your WORST level of chills or sweats during the
   past 24 hours.

| **0** | **1** | **2** | **3** | **4** | **5** | **6** | **7** | **8** | **9** | **10** |
| --- | --- | --- | --- | --- | --- | --- | --- | --- | --- | --- |
| **None** |  |  |  |  |  |  |  |  |  | **As bad as you can imagine** |

1. Please rate your **abdominal pain or bloating** by marking the one number that best describes your WORST level of abdominal pain or bloating during the past 24 hours.

| **0** | **1** | **2** | **3** | **4** | **5** | **6** | **7** | **8** | **9** | **10** |
| --- | --- | --- | --- | --- | --- | --- | --- | --- | --- | --- |
| **None** |  |  |  |  |  |  |  |  |  | **As bad as you can imagine** |

1. Please rate your **breathing symptoms** (such as shortness of breath or wheeze) by marking the one number that best describes your WORST level of breathing symptoms during the past 24 hours.

| **0** | **1** | **2** | **3** | **4** | **5** | **6** | **7** | **8** | **9** | **10** |
| --- | --- | --- | --- | --- | --- | --- | --- | --- | --- | --- |
| **None** |  |  |  |  |  |  |  |  |  | **As bad as you can imagine** |

1. Please rate your **nasal or sinus symptoms** (such as congestion or runny nose) by marking the one number that best describes your WORST level of nasal or sinus symptoms during the past 24 hours.

| **0** | **1** | **2** | **3** | **4** | **5** | **6** | **7** | **8** | **9** | **10** |
| --- | --- | --- | --- | --- | --- | --- | --- | --- | --- | --- |
| **None** |  |  |  |  |  |  |  |  |  | **As bad as you can imagine** |

1. Please rate your **skin symptoms** (such as itchiness, rash or hives) by marking the one number that best describes your WORST level of skin symptoms during the past 24 hours.

| **0** | **1** | **2** | **3** | **4** | **5** | **6** | **7** | **8** | **9** | **10** |
| --- | --- | --- | --- | --- | --- | --- | --- | --- | --- | --- |
| **None** |  |  |  |  |  |  |  |  |  | **As bad as you can imagine** |
